# Time-Lapse Incubation for Embryo Culture-Morphokinetics and Environmental Stability May Not Be Enough: Results from a Pilot Randomized Controlled Trial

**DOI:** 10.3390/jcm13061701

**Published:** 2024-03-15

**Authors:** Gilat C. Sacks, Henny Mozes, Ruth Ronn, Talia Elder-Geva, Oshrat Schonberger, Ido Ben-Ami, Naama Srebnik

**Affiliations:** 1 Shaare Zedek Medical Center, In Vitro Fertilization Unit, Jerusalem 9103102, Israel; 2 Faculty of Medicine, Hebrew University of Jerusalem, Jerusalem 9112001, Israel; taliaeg@gmail.com (T.E.-G.);

**Keywords:** time-lapse incubator (TLI), undisturbed culture, morphokinetics, embryo evaluation, environmental stability, RCT

## Abstract

**Background:** Does the Time-lapse Incubator (TLI) add value to reproductive outcomes when its two components, undisturbed culturing and morphokinetic embryo grading, are separated. **Methods:** A prospective pilot, randomized, controlled, double-blinded, single-center study was conducted during the years 2016–2020. In total, 102 patients were randomized into three groups: (1) conventional incubation with morphological evaluation only (*n* = 34), (2) TLI with both morphological and morphokinetic evaluations (*n* = 32), and (3) TLI with morphological evaluation only (*n* = 36). All arms were cultured in ESCO-MIRI^®^ incubators. A total of 1061 injected mature oocytes were evaluated (420 in arm 1, 285 in arm 2, and 356 in arm 3). The primary outcome was live birth rates. Secondary outcomes included clinical and cumulative pregnancy rates as well as embryo quality. Embryos in arm 3 were retrospectively evaluated for their morphokinetic score. **Results:** No significant difference was found in the live birth rate for single embryo transfer cycles (SET) (35% vs. 31.6% vs. 24%, *p* = 0.708) or double embryo transfer (DET) cycles (41.7% vs. 38.5% vs. 36.4%, *p* = 0.966). Comparable pregnancy rates, clinical pregnancy rates, and cumulative pregnancy were found for similar top-quality embryos for days 2, 3, and blastocyst stages across groups. A similar number of embryos were suitable for either transfer or cryopreservation within the different groups. For 62.8% of the embryos in arm 3, the morphokinetic and morphologic evaluations were similar. In only 2/36 (5.6%) treatment cycles, the use of morphokinetic scoring may have helped the patient avoid undergoing an additional treatment cycle. In the other cases, morphokinetic scoring would not have changed the end point of pregnancy. **Conclusions:** The two components of the TLI system—undisturbed culturing and morphokinetic embryo grading—do not appear to have a significant additional value in reproductive outcome, although these results should be validated by an RCT.

## 1. Introduction

The optimization of artificial reproductive technology (ART) success rates involves a combination of multiple simultaneous factors at play, while the laboratory and culture environments lay at the heart of the process. Conditions of embryo culture and embryo assessment have dramatically evolved and improved over time. Multiple professional organizations have adopted the goal of achieving singleton pregnancies should in vitro fertilization (IVF) be required [1]; hence, choosing the best possible embryo for transfer has become of prime importance.

Serial assessments of embryo morphology at specific time points and by light microscopy based on the form and structure of the embryo have been the mainstay of quality assessment in traditional incubators [2,3]. One of the primary functions of an incubator in the IVF laboratory is to create an optimal and stable environment for embryo development, with minimal variations in factors like temperature, atmospheric gas concentration, humidity, and even light exposure [4]. Still, multiple points of embryo assessment are necessary, creating unavoidable environmental disturbances. Conventional box incubators have been increasingly phased out, with the advent of multi-chamber bench-top incubators. Accordingly, the increased ability to limit disturbances in embryos during culture has resulted in improvements in embryo quality [5,6]. The introduction of time-lapse incubation has combined the most recent incubation technology with an integrated digital imaging system. This system therefore allows for the regular imaging of embryos in culture, without having to disturb the culture environment via manipulations [2,4].

The optimal time points for embryo assessment and those ‘preferential’ characteristics for embryo selection were established during the Istanbul Consensus Workshop involving Alpha Scientists in Reproductive Medicine and the European Society of Human Reproduction and Embryology (ESHRE) Special Interest Group of Embryology [3]. The notion of morphokinetics, a science combining assessment of embryo structure and form, as well as its speed of development at particular time points [7], as a predictive marker for embryo assessment, has recently materialized, with the increasing arrival of time-lapse imaging with several algorithms developed using morphokinetic parameters for embryo selection [8,9,10]. A fully automated model can reduce manual evaluation and eliminate bias due to inter- and within-observer variability [11].

Regular imaging and continuous assessment of embryos in culture have shown promise in predicting embryo development, embryo quality, and implantation potential. TLI has greatly increased our ability to detect early developmental milestones such as fertilization, cytokinesis, and karyokinesis, as well as to determine normal and abnormal cleavage dynamics [7,12]. TLI has also allowed for the discovery of aberrant morphological events that would otherwise remain unidentified [13,14,15,16].

However, a Cochrane meta-analysis, including only three RCTs on TLI culture and unpublished data, confirmed no significant difference in clinical pregnancy rate using TLI compared to the conventional incubator (OR 1.23, 95% CI = 0.96–1.59) [2]. A more recent Cochrane meta-analysis added six additional studies on this topic, this present study among the ongoing studies listed has a similar conclusion. The quality of the evidence ranged from very low to low. The main limitations were the high risk of bias in the included studies, imprecision, indirectness, and inconsistency. There were no data on cumulative live birth or ongoing pregnancy rates or cumulative clinical pregnancy rates [17]. A meta-analysis also found no significant difference in blastulation rate between TLI and non-TLI [18].

Retrospective studies reporting higher live birth rates using TLI have been limited by methodologic weaknesses, such as comparing low oxygen tension TLI culture with atmospheric oxygen tension in controls, as well as the use of different culture media in study groups [19].

Goodman et al. [20] addressed a fundamental limitation of prior studies and conducted an RCT, concluding that analysis of morphokinetic parameters did not lead to improved outcomes. The strength of this study was that both test and control embryos were incubated in TLIs. However, a third group was missing to differentiate between the TLI effects, the additional parameter of continued culturing.

To address this still-unestablished question of whether continuous time-lapse imaging and morphokinetic data improve clinical outcomes, this study was conducted. The present study was designed as a double-blinded, three-armed, randomized controlled trial to compare implantation and clinical pregnancy rates between embryos cultured in standard incubation conditions and those in TLI systems, with or without adding morphokinetic data to embryo selection criteria. All embryos were cultured in identical conditions within the MIRI ESCO incubation systems.

The objective of this study was to assess the feasibility, methodology, and potential effectiveness of a larger scale study aiming to evaluate the reproductive outcomes of the TLI system while separating the environment component and the morphokinetic embryo grading components.

## 2. Materials and Methods

### 2.1. Study Design and Population

A pilot single-center prospective randomized double-blinded study with three-arms, conducted at Shaare Zedek Medical Center, Jerusalem, Israel. Patients were recruited between November 2016 and July 2020. Trial registration number: The trial was registered at ClinicalTrials.gov Identifier: NCT02657811 on 18 January 2016.

Patients were randomized into 3 groups: (1) Benchtop incubator with morphological evaluation only, (2) TLI incubation with both morphological and morphokinetic evaluation, and (3) TLI incubation with morphological evaluation only.

The primary outcome was live birth rates. Secondary outcomes were clinical pregnancy rates and cumulative pregnancy rates.

### 2.2. Eligibility and Recruitment of Participants

Patients were eligible if they were ≤40 years of age, undergoing IVF treatment at Shaare Zedek Medical Centre, and attempting pregnancy with autologous gametes. Only patients in their first, second, or third intracytoplasmic sperm injection (ICSI) cycle were included. The cycle count was initialized from previous deliveries. Only patients with at least four mature oocytes retrieved were included. For all eligible cycles, ICSI was performed to allow standardized fertilization and exposure to TLI incubation from day 0.

Exclusion criteria included freezing all cycles, including women at high risk for ovarian hyperstimulation syndrome, uterine disorders such as untreated hydrosalpinx or abnormal endometrium, severe endometriosis, obesity (BMI > 35 kg/m^2^), the use of surgically retrieved spermatozoa, patients requiring preimplantation genetic testing (PGT), and women with low ovarian reserve (suspected to be low responders).

Upon agreement to participate, written informed consent was obtained, and patient demographic data were recorded.

**Figure 1:**
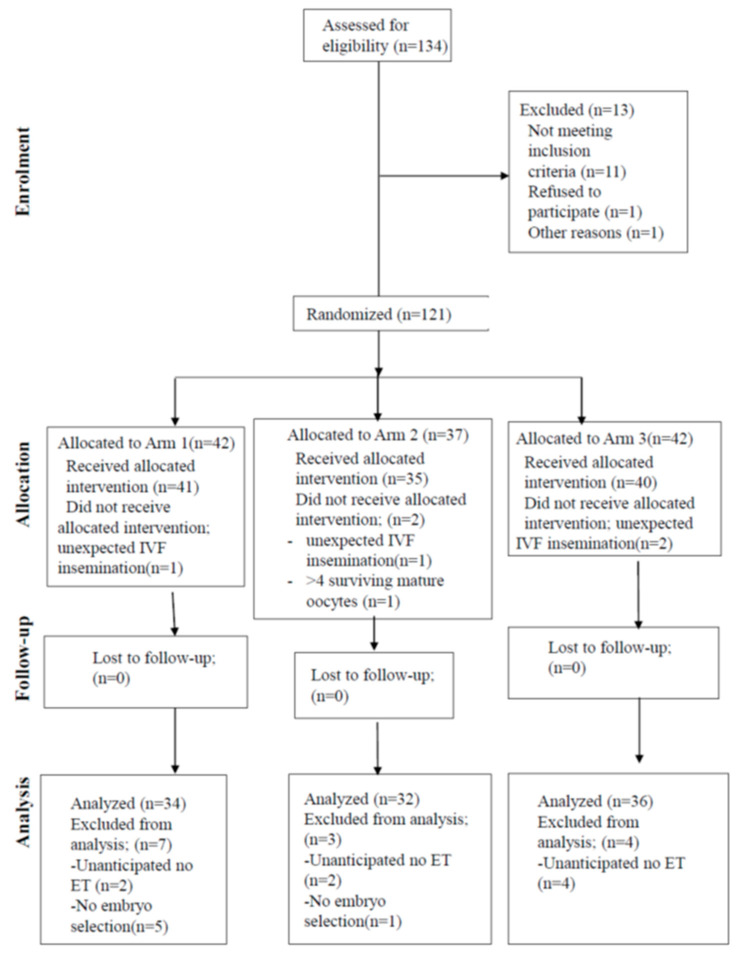
Flow chart.

Randomization was performed on the day of oocyte collection, before the ICSI procedure. Patients were randomized in a 1:1:1 ratio to either culture in the Miri Benchtop Multi-chamber incubator, Miri TLI with morphokinetic embryo evaluation, or Miri TLI with only morphological embryo evaluation. The randomization was performed using computer-generated randomization. The patient and care providers were blinded to allocation, but due to the nature of this study, it was impossible to blind the embryologist handling the embryos and performing the morphologic and morphokinetic assessments. The flowchart of the included patients is depicted in Figure 1.

Institutional review board approval was obtained. The trial was registered at ClinicalTrials.gov Identifier: NCT02657811.

### 2.3. Stimulation, Oocyte Retrieval, and ICSI

Approved study subjects underwent standard controlled ovarian stimulation (COS). Protocols and their corresponding medications were decided upon at the discretion of the treating physician. These included mainly the GnRH antagonist protocol and the long GnRH agonist protocol. Other protocols included the GnRH flare protocol (short GnRH agonist) and a combination of GnRH agonists starting at midluteal phase, followed by the GnRH antagonist protocol (agonist–antagonist protocol). Follicular aspiration was performed in the IVF unit via transvaginal ultrasound-guided needle aspiration. Endometrial preparation and luteal phase support were recommended as per the unit protocol. The day of embryo transfer and number of embryos for transfer were defined prior to the initiation of culture, according to national guidelines as mentioned above. The oocytes-cumulus complexes were denuded 2–3 h after the retrieval using hyaluronidase (Sage™ CooperSurgical^®^, Trumbull, CT, USA). ICSI was then performed on mature oocytes using droplets of polyvinylpyrrolidone (PVP) (CooperSurgical^®^ Trumbull, CT, USA) and Quinn’s Advantage™ Medium with HEPES (CooperSurgical^®^ Trumbull, CT, USA).

### 2.4. Embryo Culture

Following ICSI, the injected oocytes were then placed in culture media and loaded into preequilibrated culturing dishes (Nunc™ IVF Petri Dishes, 60 mm, Lid w/Airvent, Thermo Scientific™ Roskilde, Denmark) containing 8 droplets with 25 µL of culture medium (Global^®^Total^®^ with 5 mg/mL LifeGlobal^®^ Protein Supplement CooperSurgical^®^ Trumbull, CT, USA) covered with a 3 mL oil (LITeOil LGOL CooperSurgical^®^ Trumbull, CT, USA) overlay, cultured at 37 °C, and placed in the Esco Miri Benchtop Incubator. Embryos randomized to study arms 2 and 3 were transferred after ICSI to preequilibrated embryo dishes (CultureCoin™ Esco Medical Technologies Ltd., Kaunas, Lithuania) containing 14 individual wells with 25 µL of culture medium, as mentioned above. The dishes were covered with a 3 mL oil overlay and placed at 37 °C in the ESCO MIRI^®^ Time-Lapse Incubator (Esco Medical Technologies Ltd., Kaunas, Lithuania). Note that the environments of the Miri benchtop and Miri TL incubators were considered identical. O_2_ concentration and CO_2_ concentration were set at 5% and 5.5%, respectively.

### 2.5. Embryo Scoring and Transfer

Embryos in arm 1 of this study (Miri Benchtop Incubator) were morphologically assessed by light microscopy at pre-defined time points according to accepted criteria [3] by a trained laboratory embryologist. The time points for evaluation were at 16–18 h post insemination (HPI) (for normal fertilization), at 42 HPI (day 2), and then at 66 HPI (day 3) post-ICSI. The evaluated parameters included cell number, cell size, cell symmetry, and percent fragmentation. No gross abnormalities were noted. Embryos were graded on day 3 according to these evaluated parameters and transferred according to preferential grading (see Supplementary S1A). If embryo transfer was predetermined for day 5, additional assessment was performed at approximately 114 HPI (day 5) and 138 HPI (day 6) (see Supplementary S1 for assessment parameters).

Embryos in arm 2 of this study (Miri TL incubator) were morphologically and morphokinetically assessed by the same laboratory embryologists using digital images generated by the incubator’s integrated time-lapse imaging system. Morphokinetic and morphological assessment and scoring were performed as per the scoring classification system suggested by Desai et al. [21] and Goodman et al. [20], as adopted in our laboratory’s routine practice. Morphological screening of embryos was initially performed to discard or exclude those clearly not viable for transfer. Morphokinetic parameters were then used to rank remaining embryo score categories from a maximum of 4.0 to a minimum of −2.0, in order of hypothesized decreasing implantation potential (see Supplementary S1B).

Embryos in arm 3 of this study (Miri TLI) were morphologically assessed by the embryologists using digital images generated by the incubator’s integrated time-lapse imaging system. As noted above, the time points and evaluated parameters were identical to those in arm 1 of this study. Decisions on embryos for transfer were also identical.

For each patient, embryos were selected for transfer based on their morphologic scoring alone (Arms 1 and 3—see Supplementary S1A) or with the additional maximum morphokinetic score (Arm 2—see Supplementary S1B). The day of transfer and number of embryos for transfer were predetermined (as noted above).

Embryos were transferred an using ultrasound-guided Wallace^®^ Classic Embryo Transfer Catheter (CooperSurgical^®^, Trumbull, CT, USA) fertility companies. The attending senior physician performed the embryo transfer as per unit protocol.

Those embryos not selected but deemed appropriate for future transfer were submitted to cryopreservation by vitrification while keeping the prioritized order and score for future thawing.

When the treated cycle was completed, embryos in arm 3 were retrospectively morphokinetically graded, and the data were recorded and analyzed after this study was concluded.

Pregnancy was defined as a positive serum beta-hCG measured 14 days after embryo transfer. A clinical pregnancy was defined as the visualization of an intrauterine gestational sac with a fetal heartbeat.

### 2.6. Sample Size Calculation and Statistical Analysis

The sample size of the RCT study was calculated as such. The pregnancy rate in our IVF unit in the sub-group of patients with demographic and clinical characteristics similar to those included in this study was 40%. Under the assumption that the TLI group pregnancy rate would increase by 10%, the sample size required per group was 124 patients per arm, with an alpha risk of 5% and a power of 80%. The pilot study was planned to achieve 20% of the RCT sample size.

Data were analyzed with SPSS, version 27.0 (SPSS, Inc., Chicago, IL, USA). Data were presented as mean ± SD for continuous variables and as the number of cases or percentage for categorical variables.

The differences between the three arms of this study on maternal/patient parameters were analyzed using one-way ANOVA for continues variables and the Chi square test for categorical data.

The difference between the three arms of this study for embryo parameters was analyzed using generalized linear mixed models, which take into account the dependence of embryos (repeated measures) within each patient.

## 3. Results

### 3.1. Data on Treatment Cycles

A total of 102 women were included in the analysis after applying inclusion criteria: 34, 32, and 36 in arms 1, 2, and 3, respectively, with a total of 1061 injected mature (M2) oocytes evaluated: 420 in arm 1, 285 in arm 2, and 356 in arm 3. The basic characteristics of the groups, including age, BMI, ethnicity, and infertility diagnosis, were similar, as shown in Table 1. There was a high heterogeneity of infertility factors across the arms, although not statistically significant. Treatment protocols were similar, mostly antagonist protocols, though slightly higher rates of FSH-only gonadotropin preparations were used in group 1 (74.3% vs. 46.9%, 52.8% *p* = 0.054 for groups 1, 2, and 3, respectively), while the use of combined FSH with either recombinant LH or HCG was more prevalent in groups 2 and 3 (Table 2). The total amount of gonadotropins used was similar between the groups; however, induction was longer in arm 3 (9.2 ± 1.6 vs. 9.5 ± 1.8 vs. 10.4 ± 2.2 for groups 1,2 and 3 respectively, *p* = 0.031). Interestingly, in group 1 a larger number of oocytes (14.7 ± 5.42 vs. 10.88 ± 4.53 vs. 12.06 ± 4.59 *p* = 0.006) and M2 oocytes (11.9 ± 4.5 vs. 8.5 ± 3.6 vs. 9.6 ± 4.0 *p* = 0.005, respectively for groups 1, 2, and 3) were retrieved, and a larger number of embryos were available for evaluation (10.8 ± 4.3 vs. 8 ± 3.6 vs. 8.4 ± 3.8, *p* = 0.006); therefore, all further analyses of embryo quality and pregnancy and delivery rates were corrected to oocyte and injected M2 oocyte numbers. No differences were found in the rate of day 2, day 3, or blastocyst-stage top-quality embryos (TQE), and a similar number of embryos were suitable for either transfer or cryopreservation between the groups. The blastulation rate was calculated from 2PN embryos after the exclusion of all embryos transferred or cryopreserved on day 3. No differences were found between the groups (37.6%, 48.1%, and 42.4%, *p* = 0.085 for groups 1, 2, and 3, respectively).

The only significant finding was lower rates of multinucleation in group 1 (3.8 ± 1.6% vs. 14.7 ± 3.3% vs. 14.2 ± 3%, *p* = 0.003 for groups 1, 2, and 3, respectively), as shown in Table 3.

Regarding primary outcome, no significant difference was found in live birth rate between the groups, neither for single embryo transfer cycles (SET) (35% vs. 31.6% vs. 24%, *p* = 0.708) nor for double embryo transfer cycles (DET) (41.7% vs. 38.5% vs. 36.4%, *p* = 0.966 for groups 1, 2, and 3, respectively) (Table 4). Also, no differences were noted for pregnancy rates, clinical pregnancy rates, and cumulative pregnancy rates.

### 3.2. Morphokinetic Evaluation

There were no significant differences in the morphokinetic scoring and events between groups 2 and 3 (Table 3). Arm 3 cases were examined for the retrospective value of morphokinetic evaluation on embryo selection. In group 3, 108 embryos were either transferred (*n* = 81) or frozen (*n* = 27) in 36 ovum pick-up cycles. We compared the morphological and morphokinetic scoring of these embryos and, based on pregnancy data from frozen ET cycles, assessed the potential added value of the morphokinetic evaluation on pregnancy rates.

In 15/36 (41.7%) of the fresh ET cycles, all embryos available were transferred (22 embryos). In 9/36 (25%) cycles, all embryos transferred were concordant in their morphological and morphokinetic scores. In the remaining 12 cycles (33.3%), there was a discrepancy between the scores. In only 2/36 (5.6%) treatment cycles, the use of morphokinetic scoring may have helped the patient avoid undergoing an additional treatment cycle. In four treatment cycles, the morphological evaluation led us to transfer an embryo with a lower morphokinetic grade, but still pregnancy was achieved, while transferring the best morphokinetically graded embryo did not lead to a pregnancy, and in the other four treatment cycles, either method would have ended with the same result (pregnancy or no pregnancy).

Taking into account the frozen ET cycles, we found concordant scores in 54/86 embryos (62.8%). Logistic regression was not applied due to the small number of treatment cycles.

## 4. Discussion

This study did not identify any superiority in TLI continuous culturing or morphokinetic grading when compared to conventional incubation.

Among the three study arms, group 1 (the non-TLI arm) had a higher count of retrieved oocytes and M2 oocytes, resulting in a greater pool of embryos for assessment. Unfortunately, we could not find an explanation for the difference in the oocyte yield between the groups. The baseline parameters were similar, and randomization was applied. To prevent bias, all analyses pertaining to embryo quality, pregnancy rates, and delivery rates were adjusted based on the numbers of oocytes and injected M2 oocytes. This study observed no significant disparity in live birth rates between this study and control groups, whether considering single embryo transfer cycles (SET) or double embryo transfer cycles (DET). Furthermore, there were no differences in pregnancy rates or clinical pregnancy rates. Although based on small sample sizes, these findings prompt inquiry into the added value of the TLI system when integrating both undisturbed embryo culturing and evaluation.

The TLI technology has been extensively utilized for more than two decades for embryo culturing. However, only within the last 10 years has its capacity to complement conventional morphological assessment in embryo evaluation been investigated [12]. TLI has provided a crucial perspective into the initial stages of embryonic development, allowing us to monitor developmental landmarks and assess various abnormalities that hold predictive significance. Certain abnormalities have already been demonstrated to influence blastulation and implantation [22].

TLI creates an optimal culturing environment by minimizing handling and temperature, pH, and gas composition disturbances. Research exploring the advantages of this uninterrupted culture environment has revealed improvements in embryo quality and blastulation rates [5]. However, existing studies do not clearly prove that TLI has improved outcomes [12].

A Cochrane meta-analysis encompassing nine RCTs involving 2955 infertile couples, concluded that there is insufficient robust evidence to establish TLI’s superiority or inferiority compared to conventional embryo incubation methods [17]. The quality of the evidence ranged from very low to low due to the main limitations of high bias risk in the included studies: imprecision, indirectness, and inconsistency. There was no data on cumulative live birth, ongoing pregnancy rate, or cumulative clinical pregnancy rate. Recently, three additional studies have similarly reported uncertainty regarding the significant advantages of TLI [23,24,25].

The findings of this study did not demonstrate an advantage in embryo development within the TLI system. Notably, Group 1 (non-TLI) exhibited the lowest multinucleation rate in comparison to Groups 2 and 3, which were subjected to the TLI system. This discrepancy may be attributed to the limited time for embryo evaluation outside the incubator, leading to a lack of diagnosis. The blastulation rate varied from 37.6% in Group 1 to 48.1% in Group 2. The relatively lower rates might be attributed to the more common practice of day 3 embryo transfers in our clinic.

The initial RCT with both test and control embryos incubated in TLI systems, conducted by Goodman et al., concluded that analyzing morphokinetic parameters did not result in improved outcomes [20]. In this present study, which incorporated three study arms that all employed Miri ESCO incubators (with and without time-lapse recording), the analysis was extended to evaluate the effects of undisturbed culture and morphokinetic value by comparing arms 1 and 3 to arm 2. Nevertheless, this study did not identify any noticeable differences among the groups.

After concluding the first part of this study, embryos in arm 3 were retrospectively reevaluated, gaining additional morphokinetic data for the embryo selection process. In 62% of the embryos selected for transfer, the morphokinetic and morphologic evaluations were comparable, and only in 5.6% of the treatment cycles would the morphokinetic evaluation have contributed to the embryo selection process. In the other cases, the use of morphokinetic scoring would not have significantly changed the end point of pregnancy. In addition, the multinucleation parameter was explored for reevaluated embryos in arm 3. This was to compare its occurrence during the first morphological evaluation to the second morphokinetic observation and to strengthen the assumption that multinucleation can remain unidentified due to limited time and footage, but it was not found as such. This occurrence when comparing morphologic to morphokinetic evaluations should be further explored in a follow-up study.

This study underlines the maybe unjustified morphokinetic scoring grading system, which requires much time and attention from the embryologist to view and annotate and should be reconsidered, especially with the rise of substitute software-assisted assessment algorithms, also potentially decreasing embryologist subjectivity in embryo scoring and selection. Several algorithms have already been published [8,9,11], yet no large RCT has been published. A number of studies are currently being conducted in this area.

One of the strengths of this study lies in its meticulously designed approach, utilizing three distinct study arms to isolate the potential influences of the two components on the TLI, which may affect the outcome, the environment component, and the morphokinetic embryo grading component. A recent RCT involving 1731 couples attempted to individually assess these two components of TLI. No improvement in pregnancy outcomes was noted. Notably, a significant limitation of this research is its incorporation of 15 different clinics and 5 separate IVF laboratories using varied incubators and employing different fertilization techniques, including both slow embryo freezing and vitrification methods [25]. In our study, uniform MIRI ESCO incubators, both benchtop and time-lapse, were used, mitigating the impact of additional variables. All patients were treated in a single laboratory, adhering to strict protocols and using only the ICSI method for fertilization and only vitrification methods for freezing. This RCT was blinded to all but embryologists because of the technical nature of this study.

However, there are limitations to this study. The power calculations did not separately account for day 3 and day 5 transfers, although both were performed. Additionally, this study’s intention for single embryo transfers was at times not met due to clinical constraints. Moreover, due to the disruption caused by the COVID-19 pandemic, patient recruitment was hindered, leading to study termination. Despite this limitation, this study identified a significant association with the multinucleation parameter. Given the TLI system’s capacity to provide extended observation time and footage, this finding was expected, enabling the detection of abnormal cleavage dynamics. Furthermore, the substantial number of evaluated embryos facilitated conclusions regarding their development. Importantly, no differences were observed in terms of top-quality embryos across the different groups. Another limitation is the high heterogeneity of infertility factors across the arms, although they are not statistically significant. This could not be controlled since randomization was performed, and these factors fell within the inclusion criteria. Another limitation is the lack of semen parameters, but ICSI was performed in all patients.

## 5. Conclusions

This study aimed to evaluate separately the potential added value of the two components of the TLI: uninterrupted culture and the morphokinetic grading system, using uniform incubation systems for all three arms of this study. Despite this study’s limited sample size, the results suggest that the TLI system alone does not offer significant additional value. Our study results should be validated in a larger-scale RCT.

## Figures and Tables

**Table 1:** Basic characteristics of this study population.

	Non-TLI Morphology Only *n* = 34	TLI Morphokinetics *n* = 32	TLI Morphology Only *n* = 36	*p*-Value
Arm	1	2	3	
Age	32.4706 ± 4.6399	33.9063 ± 5.0119	32.2778 ± 4.5205	0.313
	8	8	0	
BMI	23.7457 ± 4.7760	23.4653 ± 3.9358	25.1597 ± 5.8865	0.338
	9	1	2	
Ethnicity Jewish	31 (88.6%)	31 (96.9%)	34 (84.4%)	0.376
Ethnicity Arab	4 (11.4%)	1 (3.1%)	2 (5.6%)	
IVF indication				
Unexplained	17 (50%)	23 (71.9%)	18 (50%)	0.472
Ovulatory	1 (2.9%)	1 (3.1%)	1 (2.8%)	
Male factor	10 (29.4%)	3 (9.4%)	9 (25%)	
Mechanical	1 (2.9%)	3 (9.4%)	3 (8.3%)	
Mixed	5 (14.7%)	2 (6.3%)	5 (13.9%)	

**Table 2:** Treatment cycle data.

	Non-TLI Morphology Only *n* = 34	TLI Morphokinetics *n* = 32	TLI Morphology Only *n* = 36	*p* Value
Induction protocol				
GnRH antagonist	31 (88.6%)	25 (78.1%)	24 (68.6%)	
Long GnRH agonist	4 (11.4%)	4 (12.5%)	7 (20%)	
Other	0 (0%)	3 (9.4%)	4 (11.5%)	0.322
Type of gonadotropins				
FSH only	26 (74.3%)	15 (46.9%)	19 (52.8%)	0.054
Combined LH and FSH	9 (25.7%)	17 (53.1%)	17 (42.2%)	
Total gonadotropin dose (IU)	1985.27 ± 1275 31	2120.83 ± 1084 32	2025.59 ± 1084 25	0.887
Days of stimulation	9.2 ± 1.6	9.5 ± 1.8	10.4 ± 2.2	0.031
Maximum E2 level (pmol/L)	5972 ± 2992.8	5038 ± 2055	5376 ± 2397	0.312
Oocyte number	14.7 ± 5.42	10.88 ± 4.53	12.06 ± 4.59	0.006
M2 oocytes	11.9 ± 4.5	8.5 ± 3.6	9.6 ± 4.0	0.005
Fertilization rate (%)	82.7 ± 20.3	85.0 ± 17.2	83.0 ± 17.9	0.859
Number fertilized	9.7 ± 4	7.2 ± 3.4	7.6 ± 3.3	0.014
Number of embryos per patient	10.8 ±4.3	8 ±3.6	8.4 ± 3.8	0.006
Day 2 top quality rate (%)	65.3 ± 4.4	63.6 ± 4.2	64.5 ± 4	0.92
Day 3 top quality rate (%)	65.3 ± 5	60.8 ± 4.9	61.4 ± 4.5	0.857
Blastulation rate (%)	37.6	48.1	42.4	0.085
Top quality blastocyst rate (%)	17.3 ± 3	23.7 ± 3.7	17.9 ± 3	0.421
MU multinucleation (%)	3.8 ± 1.6	14.7 ± 3.3	14.2 ± 3	0.003
Embryo ET/cryopreserved rate (%)	32.4 ± 3.3	38.4 ± 3.8	30.9 ± 3.3	0.418

**Table 3:** Morphokinetic evaluation.

Embryo Data	Corrected for Embryo and Oocytes Number			
	Non-TLI Morphology Only *n* = 420	TLI Morphokinetics *n* = 285	TLI Morphology Only *n* = 356	*p*-Value
Morphokinetic score		0.354 ± 0.08	0.37 ± 0.07	0.89
t5		51.76 ± 1.02	50.43 ± 0.9 1	0.33 3
tSB		102.51 ± 1.22	103.4 ± 1.1 1	0.58 9
cc2		9.584 ± 0.989	8.562 ± 0.9 02	0.45 7
s2		3.776 ± 1.05	4.34 ± 0.96	0.66 4
s3		1.676 ± 2.84	3.19 ± 2.62	0.66
MU	3.8 ± 1.6	14.7 ± 3.3	14.2 ± 3	0.00 3
RCLV		0.175 ± 0.06	0.220 ± 0.0 8	0.63 5
DUC		0.281 ± 0.09	0.514 ± 0.1 2	0.12 2
ID		0.114 ± 0.06	0.173 ± 0.0 9	0.57 2

MU = multinucleation; RCLV = reverse cleavage; DUC = direct uneven cleavage; ID = irregular division.

**Table 4:** Treatment outcome.

	Non-TLI Morphology Only *n* = 34	TLI Morphokinetics *n* = 32	TLI Morphology Only *n* = 36	*p*-Value
SET transfer data	*n* = 22	*n* = 19	*n* = 25	
Positive bHCG	13 (59.1%)	10 (52.6%)	9 (36%)	0.262
Clinical pregnancy	11 (50%)	6 (31.6%)	6 (24%)	0.164
Live birth	7 (35%)	6 (31.6%)	6 (24%)	0.708
DET transfer data	*n* = 12	*n* = 13	*n* = 11	
Positive bHCG	6 (50%)	6 (46.2%)	5 (45.5%)	0.972
Clinical pregnancy	5 (41.7%)	5 (38.5%)	4 (36.4%)	0.966
Live birth	5 (41.7%)	5 (38.5%)	4 (36.4%)	0.966
Cumulative Live birth rate	73.5%	68.8%	63.9%	0.182

## Data Availability

Data is unavailable due to privacy and ethical restrictions.

## Supplementary Materials

The following supporting information can be downloaded at: https://www.mdpi.com/article/10.3390/jcm13061701/s1.
