# Supplementary material for: Time-Lapse Incubation for Embryo Culture-Morphokinetics and Environmental Stability May Not Be Enough: Results from a Pilot Randomized Controlled Trial
Source: J Clin Med. 2024 Mar 15;13(6):1701. doi: 10.3390/jcm13061701 (PMC10971676; doi:10.3390/jcm13061701)
Supplement: Supplementary file 1 [file jcm-13-01701-s001.zip › jcm-2813509-supplementary.pdf]

Supplementary S1: Morphologic and Morphokinetic grading Criteria and scoring system

Supplementary S1A:Morphologic grading Criteria for embryos from all Arms (1 to 3) of the study:

Time Points of assessment: (as per Desai 2014 as seen in Goodman 2016)

- 16 – 18 HPI
- 42 HPI (d2)
- 66 HPI (d3)
- +- 90 HPI
- 114 HPI
- +- 138 HPI

<u>Overall Evaluated morphologic parameters:</u>

- oocyte Polar body presence?
- 2PN post-fertilization?
- cell number (Optimal?)
- Cell size & symmetry
- percent fragmentation.
- Multinucleation
- +- Blastocyst check – Optimal parameters:
    - Stage of development: Early blastocyst, Blastocyst, Expanded, Hatched/Hatching
    - Inner Cell Mass (ICM) grade: A,B, C
    - Trophectoderm (TE) grade: A, B, C

Breakdown of assessment:

**Pre-fertilization**	PB presence?			
**16 - 18 HPI**	2 PN?			
**42 HPI (d2)**	# Cells?	% Fragmentation?	Multinucleation?	Appropriate cell size / symmetry?
**66 HPI (d3)**	# Cells?	% Fragmentation?	Multinucleation?	Appropriate cell size / symmetry?
**+- 90 HPI (morula)**	Stage?	Inner Cell mass (ICM) grade?	Trophectoderm (TE) grade?	
**114 HPI (exp blast)**	Stage?	Inner Cell mass (ICM) grade?	Trophectoderm (TE) grade?	
**+- 138 HPI (hatched blast)**	Stage?	Inner Cell mass (ICM) grade?	Trophectoderm (TE) grade?	

Optimal Parameters:

- <u>Day 0 check: oocyte parameters:</u>
    - Examination of polar body presence
- <u>Day 1 / fertilization check:</u>
    - 2 Pronuclei present
- <u>Day 2 check:</u>
    - Four cells

    - Minimal fragmentation (<10%)
    - No multinucleation
    - Symmetry at 4 cells
- Day 3 check:
    - Eight cells
    - Minimal fragmentation (<10%)
    - Symmetry at 8 cells
- Blastocyst check:
    - Stage of development: Early blastocyst, Blastocyst, Expanded, Hatched/Hatching
    - Inner Cell Mass (ICM) grade:
        - A (Good): Prominent, easily discernible, with many cells that are compacted and tightly adhered together
        - B (Fair): Easily discernible, with many cells that are loosely grouped together
        - C (Poor): Difficult to discern, with few cells
    - Trophectoderm (TE):
        - A (Good): Many cells forming a cohesive epithelium
        - B (Fair): Few cells forming a loose epithelium
        - C (Poor): Very few cells

- **Embryos graded on day 3 or day 5 (predetermined) and transferred according to preferential morphological grading (if in Arm 1 or 3)**

Supplementary S1B: **Morphokinetic grading Criteria and scoring system**

Morphokinetic Criteria recorded for ALL embryos in TLI (Arm 2&3):

(t0 = time of insemination)

- **tPNf**: time to pronuclear fading
- **t2**: time to 2 cells
- **t3**: time to 3 cells
- **t4**: time to 4 cells
- **t5**: time to 5 cells
- **t8**: time to 8 cells
- **t9**: time to 9 cells
- **tMor**: time to morula
- **tSB**: time to start of blastulation
- **tBL**: time to blastocyst
- **tEBL**: time to expanded blastocyst
- **cc2**: duration of the 2nd cell cycle (t3-t2)
- **cc3**: duration of the 3rd cell cycle (t5-t3)
- **t4int**: interval between 4 and 5 cells (t5-t4)
- **t5-t2**: interval between 2 cells and 5 cells
- **s1**: t2-tPNf
- **s2**: t4-t3
- **s3**: t8-t5

Other qualitative Data:

- **Multinucleation (MU):**
  - more than one nuclei in a cell observed in 2–4-cell-stage embryos
  - recorded based on the number of cells affected and the number of nuclei per cell
- **Reverse Cleavage (RCLV):**
  - reabsorption of a blastomere after previous visual confirmation.
- **Direct uneven cleavage (DUC):**
  - evidence of one blastomere directly splitting to three
- **Irregular Division (ID)**

  1→3 Direct or rapid (>5h) cleavage from 1 to 3 cells, referred to as irregular division pattern
  - 2→5 Direct or rapid (>5h) cleavage from 1 to 3 cells, referred to as irregular division pattern

Morphokinetic Criteria,additional grading for Embryos from Arm 2 (and Arm 3 retrospectively):

HPI = Hours post-insemination

- POSITIVE points:
  - t5 = 45.8–57.0h HPI (+1)
  - s2 = 0-0.1h (+1)
  - s3 = 1.4-7h (+1)
  - tSB < 100 HPI (+1)
- NEGATIVE points:
  - cc2 <5h (-1)
  - Multinucleation (-0.5)
  - Irregular division (-0.5)

- SCORE=________

Embryo initially screened by morphology

THEN embryos with the highest morphokinetic score (MAX of 4 and MIN of -2.0) selected for transfer.
